# Membrane Systems for Tissue Engineering 2020

**DOI:** 10.3390/membranes11100763

**Published:** 2021-10-01

**Authors:** Sabrina Morelli, Shih-Jung Liu, Loredana De Bartolo

**Affiliations:** 1 Institute on Membrane Technology, National Research Council of Italy, Via P. Bucci, Cubo 17/C, I-87036 Rende, CS, Italy; l.debartolo@itm.cnr.it; 2 Department of Mechanical Engineering, Chang Gung University, Taoyuan 33302, Taiwan; shihjung@mail.cgu.edu.tw

Membrane systems offer a broad range of applications in the field of tissue engineering. Due to their peculiar selective properties, polymeric membranes provide the biofunctionality, mechanical, and topographical features of the native target tissue, enhancing the repair and regeneration of injured tissues/organs. Pioneering works in this domain have validated membrane tools as reliable investigational platforms that are able to create morpho-functional analogues of tissues and organs for studying specific biological phenomena in normal conditions and for in vitro disease modelling to disclose the therapeutic potential of new molecules.

This Special Issue (SI) highlights the multifunctional role of membrane systems for tissue engineering applications and combines original experimental works with a review prepared by renowned experts. Eleven papers were published that cover the main trends and challenges, actual applications, and potentiality of membranes in such fields of research.

The paper by Chen et al. [1] addresses the issue that most natural water-soluble polymers are difficult to electrospin due to their specific chain conformation in aqueous solution, which limits their applications. They established an entanglement model to explain the effect of polyethylene oxide (PEO) on the electrospinnability of hyaluronic acid (HA) in aqueous solutions to determine the factors that affect the electrospinnability of water-soluble polymers. PEO with a relatively high molecular weight with limited crystal formation formed extensive chain entanglements with HA, while PEO with relatively low molecular weight weakened the interactions among HA chains. These findings provide useful information for obtaining natural polymer nanofibers and are helpful for understanding the electrospinning processes of other natural polyelectrolytes to be used in tissue engineering.

Three papers are focused on the development of a new class of hollow fibers (HFs) called microtube array membranes (MTAMs) for cancer treatment [2], as hemoperfusion devices [3], and as vascular grafts [4]. MTAMs consist of ultra-thin, one-to-one connected microtube fibers that are arranged in an arrayed parallel formation. Compared to traditional HFs, the lumen walls of MTAMs are 100 times thinner than that of traditional HFs.

A novel electrospun polysulfone (PSf) MTAM, possessing excellent diffusion, surface area, and biocompatibility, was developed and proposed as a novel approach for immunotherapy-based anti-cancer treatment [2]. Hybridoma cells were encapsulated within the PSf MTAMs and successfully survived and continuously released sufficiently high levels of specific antibodies that suppressed cancer cells. The work shows that the PSf MTAMs were not only an excellent three-dimensional (3D) cell culture substrate but that they can be applied as an implantable and more importantly removable system for encapsulated cell therapy.

To further improve the capability of MTAMs, Chen et al. [3] successfully developed the next generation MTAMs, which has a superior surface area that is up to 2.3–2.5 times that of traditional MTAMs. Three distinctive variants of MTAMs were obtained by tri-axial electrospinning, namely the traditional single-layered PSF MTAM, the alternating-layered MTAM (PSF MTAM-A) with trapezium-shaped lumens arranged in an alternating arrayed formation, and the single-layered MTAM with hairy nanostructures. Among these MTAM variants, the novel PSF MTAM-A with the greatest surface area and packing density was selected for the downstream immobilization of polymyxin B (PMB) and endotoxin absorption testing. The paper emphasizes the potential employment of such MTAM-A-PMB as a hemoperfusion device for the removal of endotoxins.

With the aim of realizing small diameter blood vessels, another kind of MTAMs was fabricated by co-axial electrospinning using poly lactic-co-glycolic acid (PLGA) [4]. Compared to previous MTAMs, such novel electrospun membranes have unique characteristics, including the high porosity of the ultra-thin lumen wall with a very homogenous pore distribution, suggesting the excellent ability to precisely control pore size through the porogen–surfactant ratio. The fibers appeared to be square in shape, had good flexibility, and could be easily manipulated, allowing them to be rolled into a tubular form. PLGA MTAMs were successfully used for the co-culture of smooth muscle cells and human endothelial vein cells, suggesting their potentiality for applications as tissue engineered vascular grafts, and they could also be used for coronary artery bypass surgeries.

In the field of bone tissue engineering/regeneration, the papers of Bassi et al. [5,6] aimed to evaluate the efficiency of different kind of biocompatible and biodegradable membranes in the bone repair of 8-mm critical size defects in rat calvaria. Bacterial cellulose membranes (Nanoskin^®^) (BC) showed interesting properties and presented excellent results in the repair of large soft-tissue injuries [5]. Nevertheless, they showed low biocompatibility in critical defects in rat skulls when compared to collagen membranes (Bio-Guide^®^—positive control—BG), which better supported bone regeneration than the BC membranes. To improve the performance and the biodegradable characteristics of the BC membranes, the authors suggest various strategies, such as enzyme embedding and protein incorporation.

Polycaprolactone (PCL) membranes enriched with 5% hydroxyapatite presented good histological results due to the addition of hydroxyapatite and its osteogenic and osteopromotive factors [6]. Interestingly, it induced similar bone healing, which was observed in the control group (BG membranes), showing complete closure of the critical defect. Thus, the PCL membrane displayed good potential for guiding bone regeneration.

A big part of this SI is dedicated to membrane-based systems as tissue/organ models. In such models, polymeric membranes are mostly used as supports on which cells are cultured to create functional tissue units for the desired organ. To this end, the membrane properties, e.g., morphology and porosity, should match the tissue properties. Organ models of dynamic (barrier) tissues, e.g., lung, require flexible, elastic, and porous membranes. Thus, membranes based on poly (dimethyl siloxane) (PDMS) are often applied, which are flexible and elastic. However, PDMS has limited cell adhesive properties and displays small molecule ad- and absorption. Furthermore, the introduction of porosity in these membranes requires elaborate methods. Using evaporation-induced phase separation (EIPS), Pasman et al. [7] developed a new method in this field that is based on solvent evaporation initiating phase separation followed by membrane photo-crosslinking, resulting in new porous membranes for organ models based on poly (trimethylene carbonate) (PTMC). They tailored the PTMC membrane morphology and porosity through a detailed investigation of various parameters, including polymer dope additives, humidity, and the type and amount of non-solvent. The resulting membranes showed high water permeance that was similar or higher than that of commercial membranes, good mechanical properties, and suitable pore sizes for application in in vitro organ models. Moreover, the obtained understanding of EIPS and its established compatibility with photo-crosslinking could be used to prepare membranes from other amorphous or semi-crystalline polymers for biomedical applications.

Novel in vitro drug-screening platforms mimicking cardiac tissue properties are essential for drug discovery in cardiovascular disease. Allijn et al. [8] hypothesize that the combination of PTMC and poly (ethylene glycol) (PEG) could result in highly tunable membranes, with Young’s moduli in the physiological and pathological cardiac tissue range. This will enable drug efficacy studies on cardiomyocytes (CMs) in both healthy and diseased conditions. They prepared six thin and transparent membranes based on methacrylate-functionalized macromers of PTMC, PEG, and the triblock-copolymer PTMC-PEG-PTMC. Among these substrates, the PTMC10:PEG90 membranes had a stiffness that was close to that of physiological cardiac tissue and was still tough enough for handling and upscaling. Furthermore, this membrane exhibited very low verapamil adsorption, and the CMs showed healthy contraction behavior. Therefore, it is a good candidate for the in vitro mimicry of cardiac tissue for drug screening applications.

Current challenges in the field of neuronal tissue engineering include the creation of in vitro membrane-based models of brain tissue by combining neurons, membranes, and therapeutic molecules, which could be used as innovative approaches to predict the results of in vivo studies and provide therapeutic strategies enhancing neuronal regeneration. This SI includes two interesting studies on the realization of advanced membrane-based tools that have been employed as in vitro models of the blood–brain barrier (BBB) [9] and neurological diseases [10], for studying CNS drug delivery efficiency, and for testing pharmaceutical compounds in neurodegenerative diseases.

Montecon-Oria et al. [9] developed PCL and composite PCL/graphene (PCL/G) HF membranes using the phase inversion technique for the realization of an in vitro BBB model to test the permeability of innovative drugs for treating neurological disorders. Both kind of HFs have morphological and mechanical properties that enable their use as scaffolds for cell culture in BBB models. The presence of graphene in PCL/G membranes enlarged the pore size and the water flux and presented significantly higher electrical conductivity than PCL HFs. Particularly, this study indicates the potential of PCL HF membranes to grow endothelial cells and PCL/G HF membranes to differentiate astrocytes, the two characteristic cell types that could develop in vitro BBB models in future 3D co-culture systems.

Interestingly, Piscioneri et al. [10] present the successful development of a multiplex membrane device that consists of a continuous array of PLGA membrane chambers as an innovative platform for performing different and simultaneous investigations. Morphological, structural, physico-chemical, and mechanical properties of PLGA membranes support cell adhesion, growth, and neuronal differentiation. The PLGA multiplex membrane platform enabled the demonstration of the role of trans-crocetin double protective action in defending neurons against both oxidative stress and Aβ-induced toxicity, which play important roles in the pathogenesis of Alzheimer’s disease. Therefore, the multiplex membrane system offers precise and reliable cell responses and serves as an easy handling platform for the initial and fast screening of therapeutic compounds in controlled conditions.

A review paper completes this SI [11]. It summarizes the recent advances and trends on the development of membrane scaffolds from synthetic polymers and hybrid materials employed for the engineering of cartilage tissue and regenerative medicine. 

Given these diverse contributions, it is evident that the development of polymeric membrane systems applied to tissue engineering is a growing research area that is under continuous evolution that will lead to innovative solutions in this field. We hope that this SI not only provides a source of information but that is can also serve to stimulate new ideas. Finally, as guest editors, we would like to sincerely thank all of the authors, reviewers, and the publisher for their outstanding work.
